# Implementation of VMAT‐based lattice spatially fractionated radiation therapy (SFRT) across linac platforms: Practical considerations for QA and motion management

**DOI:** 10.1002/acm2.70695

**Published:** 2026-07-10

**Authors:** Somayeh Gholami, Prema Rassiah

**Affiliations:** ^1^ Department of Radiation Oncology Huntsman Cancer Institute University of Utah Salt Lake City Utah USA

**Keywords:** peak‐to‐valley, SFRT implementation, SFRT motion management, SFRT QA, VMAT Lattice SFRT

## Abstract

**Background:**

Spatially fractionated radiation therapy (SFRT) offers a method to treat bulky, radioresistant tumors while sparing normal tissues. Modern volumetric modulated arc therapy (VMAT) delivery enables conformal lattice SFRT plans, but practical guidance for implementation across different linear accelerators is limited.

**Purpose:**

This study presents a structured clinical workflow for implementing lattice SFRT across Edge and Halcyon linacs, with emphasis on quality assurance (QA) and motion management.

**Methods:**

A systematic workflow was developed using Eclipse TPS (Acuros v16) with 6 MV flattening filter–free (FFF) beams and applied to Edge and Halcyon linacs. The implementation progressed from 28 preliminary phantom and test plans to 12 representative clinical test plans across head and neck, pelvis, and extremity sites, and 6 lung plans evaluating respiratory motion effects with and without the SDX breath‐hold system. Two prescription strategies were investigated: (a) a single‐fraction lattice regimen delivering 18 Gy to vertices with valley doses < 6 Gy, and (b) a simultaneous integrated boost (SIB) approach delivering 66.7 Gy to vertices with 20 Gy in 5 fractions to the entire target. Dosimetric accuracy and deliverability were evaluated using Delta4, Electronic Portal Imaging Device (EPID), and film measurements.

**Results:**

VMAT‐based lattice SFRT plans were successfully delivered on both linacs with consistent dosimetric performance. Point‐based peak‐to‐valley dose ratios (PVDRs) ranged from 2.8 to 3.4 across the two platforms for both dose prescriptions. Delta4 and EPID gamma pass rates exceeded 90% (3%/1 mm), and film dosimetry agreed within ∼5% uncertainty. Comparable outcomes between Edge and Halcyon support cross‐platform reproducibility. In the evaluated lung cases, motion on the order of 5 mm was associated with changes in organ‐at‐risk (OAR) doses, highlighting the need for motion management.

**Conclusions:**

This structured framework demonstrates that lattice SFRT can be safely implemented across different linac platforms. While dosimetric outcomes are institution‐dependent, the method provides practical guidance for clinical adoption.

## 1 INTRODUCTION

In recent years, there has been a resurgence of interest in SFRT, particularly in the context of modern radiotherapy techniques.^1^ SFRT is not a new concept; it has its historical roots in grid therapy, which was developed decades ago as a means to treat bulky, often radioresistant tumors that were difficult to control with conventional uniform‐dose irradiation.^2^, ^3^ Grid therapy traditionally used physical blocks or collimators to shape the beam into a pattern of high‐ and low‐dose regions, referred to as peaks and valleys.^2^, ^4^ However, the limitations of 2D planning and the use of heavy physical blocks restricted its use.^5^

Advances in imaging and treatment delivery have reestablished the clinical feasibility of spatially fractionated approaches. High‐resolution CT, 4DCT, and MRI enable improved target and OAR delineation, while modern inverse planning algorithms and MLC‐based delivery systems allow accurate modeling of steep three‐dimensional dose gradients.^6^ Although grid‐like dose patterns have been produced using static IMRT, prolonged treatment times and delivery inefficiencies have limited its practicality. In contrast, VMAT enables efficient delivery of lattice SFRT, in which discrete high‐dose vertices are embedded within the tumor volume and separated by low‐dose valleys. This approach eliminates the need for physical grid blocks and allows treatment of deep‐seated or irregularly shaped tumors with improved conformity.^7^

However, lattice SFRT introduces technical challenges distinct from conventional VMAT planning. Unlike uniform‐dose treatments, lattice SFRT deliberately creates steep intratumoral dose gradients, requiring careful consideration of contouring strategies, optimization parameters, and dosimetric metrics such as the PVDR. In addition, respiratory motion can significantly impact dose distributions in the presence of steep dose gradients, making motion management an important consideration for clinical implementation.^8^

Despite growing clinical interest, there is currently limited consensus and no widely standardized guidelines for VMAT‐based lattice SFRT planning, commissioning, or quality assurance (QA).^9^, ^10^ Addressing this gap requires a structured framework that integrates contouring approaches, planning strategies, dosimetric verification, and motion management considerations. To translate these conceptual requirements into a clinically usable process, it is essential to evaluate the framework across different delivery systems to assess feasibility and reproducibility.

In this study, two linac platforms, Edge TrueBeam and Halcyon (Varian, Palo Alto, CA)—with distinct MLC designs and delivery characteristics were evaluated. Although both systems had been previously commissioned for standard clinical use, lattice SFRT necessitates additional technique‐specific validation prior to patient treatment.

The goal of this work is to present a practical, workflow‐based approach for implementing lattice SFRT rather than to define a single, universally reproducible planning solution. Because lattice SFRT outcomes are influenced by linac MLC design, treatment planning system, and prescription strategy, dosimetric results are inherently institution‐dependent. Accordingly, this study emphasizes a stepwise implementation process, from phantom testing to representative clinical scenarios, incorporating measurement‐based QA and motion analysis to provide a flexible roadmap for safe clinical adoption.

## 2 METHODS

To establish a systematic process for lattice SFRT implementation, multiple plans with varying levels of complexity—from single‐vertex to multi‐vertex lattice arrangements—were first designed and tested in phantom. These were considered preliminary tests. Subsequently, lattice plans for different anatomical sites were created using previously treated patient CT data sets to verify the findings from the preliminary phase. All planning and dosimetry evaluations were performed on two different treatment platforms, Edge TrueBeam and Halcyon (Varian, Palo Alto, CA), to assess the reproducibility of the proposed workflow across machines. These systems differ substantially in MLC design, with Edge employing a high‐definition MLC (2.5 mm central leaf width at isocenter), whereas Halcyon utilizes a dual‐layer stacked and staggered MLC with an effective 5 mm leaf resolution, resulting in distinct modulation and dose‐shaping characteristics.^11^

### 2.1 Plan design and parameters

Treatment planning was performed using the Eclipse TPS (Varian Medical Systems, Palo Alto, CA) with the Acuros v16 dose calculation algorithm, chosen for its advanced modeling of scatter and heterogeneity effects.^12^

A 6 MV FFF photon beam was selected for delivery due to its higher dose rate and sharper penumbra, which are critical for maintaining steep dose gradients. A high‐resolution dose grid (1 ‐1.25 mm) was employed to minimize volume averaging. IMRT optimization objectives for OARs were based on SBRT guidelines,^13^ with adjustments according to fractionation schemes.

### 2.2 Prescription regimens

In SFRT, the prescribed doses typically range from 10–20 Gy in a single or in a fractionated regimen to deliver up to 66.7 Gy to the high‐dose vertices while delivering 20 Gy in 5 fractions^14^ to the gross tumor volume (GTV), followed by conventional radiation therapy. For this study, two dose‐prescription strategies, based on previous clinical implementations for each anatomical site, were evaluated:

1. Single‐fraction regimen: 18 Gy delivered to peak (vertex) regions, with valley doses kept below 6 Gy.
2. Fractionated regimen: A simultaneous integrated boost approach, delivering 66.7 Gy to vertices and 20 Gy in 5 fractions to 95% of the GTV

### 2.3 Preliminary tests

Preliminary tests were conducted as a phantom‐based study. Different GTV sizes were generated in a solid‐water phantom. The SFRT GTV was then defined by shrinking the conventional GTV by 5 mm to centrally confine the vertices and avoid the tumor periphery. Vertices were arranged in a lattice pattern with diameters of 0.5–1 cm, center‐to‐center spacing of 3–5.5 cm, and inter‐plane separation of approximately 1.5–2 cm. The ratio of vertex tumor volume (VTV) to the SFRT GTV was kept below 1% to balance high‐dose focality with overall tumor sparing.^8^, ^15^ First, a single‐vertex contour was evaluated to ensure the creation of localized high‐dose regions with steep peak‐to‐valley gradients. Subsequently, plans with 2, 3, and 7 vertices in a single axial plane were assessed using vertex diameters of 0.5 and 1 cm and center‐to‐center spacing of 3–5.5 cm.

**FIGURE 1:**
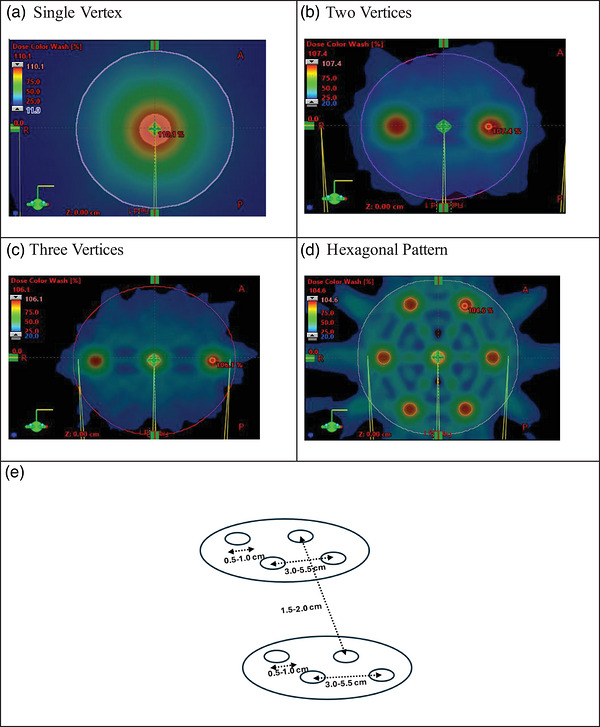
Dose distributions for: (a) single vertex, (b) two vertices, (c) three vertices, (d) hexagonal pattern, and (e) schematic diagram of the vertex pattern and interplanar distance.

Finally, the number of vertices and planes was increased up to 50 vertices distributed across 3–4 planes, to simulate clinically realistic three‐dimensional lattice patterns. One to four arcs with collimator rotations (e.g., 10°–20°) were selected depending on plan complexity and vertex count. When necessary, manual normal tissue optimization (NTO) was applied, and customized optimization contours were introduced to enhance dose‐gradient sharpness (Figure 1).

Smaller vertices produced sharper gradients and higher PVDR but posed greater challenges in MLC positioning and delivery reproducibility. Larger vertices were easier to deliver but tended to reduce peak‐to‐valley distinction. Therefore, depending on GTV size, the number of vertices and optimization parameters, the lattice spacing was adjusted to maintain valley doses within ± 10% of the prescribed valley dose. After achieving the desired plan for each geometry and vertex size on the Edge machine, the vertex spacing was systematically increased in 5 mm intervals to obtain comparable dose distributions and PVDR on the Halcyon machine. Plan normalization was adjusted to maintain the maximum absolute dose within 1% between corresponding plans across the two machines.

For PVDR evaluation, both a DVH‐based surrogate (D10% ÷ D90% for the target)^16^ and a point‐based metric defined as the ratio of the dose at the vertex center to the dose at the midpoint between adjacent vertices, derived from dose distributions in the axial, sagittal, and coronal planes, were considered. A Halcyon plan was considered acceptable if the variation in point‐based PVDR between platforms was within ± 10% and the DVH‐based PVDR remained within ± 2%.  This tolerance is due to measurement sensitivity and spatial sampling limits, not a direct comparison between planned and achieved PVDR values. A larger tolerance was accepted for the point‐based PVDR due to its higher sensitivity to spatial sampling and resolution.

Across both the Edge and Halcyon platforms, a total of 28 preliminary plans were generated, accounting for different vertex sizes and separations.

### 2.4 Clinical plan tests

Six representative clinical plans were generated using available patient CT datasets, including two cases each from the head and neck, pelvis–abdomen, and extremity. These anatomical sites were selected to capture common clinical challenges, including proximity to critical OARs, large target volumes, and peripheral tumor locations.

**FIGURE 2:**
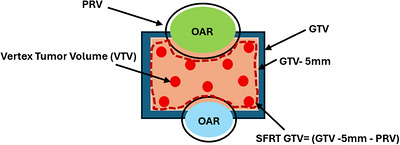
A simple schematic showing different volumes considered for SFRT clinical tests.

For clinical plans in which OAR contours were available, a 5‐mm planning organ‐at‐risk volume (PRV) margin was applied to all OARs for safety. All target and OAR contours were manually reviewed to ensure appropriate conformity to the GTV and to avoid overlap between SFRT vertices and OARs, as illustrated in Figure 2.

In addition, three lung CT cases with respiratory motion exceeding 5 mm were evaluated, resulting in six lung SFRT plans generated across both platforms (Edge and Halcyon). CT scans were acquired with and without the SDX Respiratory Gating System (DYN'R Medical Systems, Toulouse, France), which employs a breath‐hold–based motion‐management approach.^17^ For each case, plans were generated on both Edge and Halcyon platforms. Two motion‐management strategies were assessed: (1) using a mean CT from the 4D dataset, with the SFRT GTV derived from the internal target volume (ITV) plus a 5 mm inner margin, and (2) using the SDX breath‐hold CT to define the SFRT GTV directly from the breath‐hold GTV with a 5 mm inward margin. To evaluate the dosimetric impact of motion, SFRT GTV contours and vertex geometries defined on the mean CT were propagated onto the SDX CT, and OAR doses were recalculated to simulate conditions without motion mitigation.

All clinical plans were generated on both the Edge and Halcyon platforms, resulting in a total of 18 clinical plans.

### 2.5 Dosimetric verification

For all plans, IMRT QAs were performed using Delta4 Phantom+ (ScandiDos, Sweden) and EPID.

Gafchromic EBT‐XD film dosimetry (Ashland ISP Advanced Materials, NJ, USA), batch # 10172301, was used for dosimetric measurements for the preliminary tests due to its high spatial resolution and suitability for high‐dose applications (up to ∼40 Gy), as reported in the literature.^18^ The films were calibrated using known dose exposures ranging from 0.5 to 25 Gy in 100 cGy increments. Calibration was performed using a multichannel (red, green, blue) analysis method to reduce dosimetric uncertainties. All films were scanned using a 48‐bit (16‐bit per channel) flatbed scanner (Epson Expression 11000XL) at 72 dpi in transmission mode, with consistent orientation and scanning conditions. Both calibration and measurement films were scanned 48 hours post‐irradiation to ensure signal stabilization. Calibration, scanning, and analysis were performed using MATLAB 7.9.0 (MathWorks, Natick, MA), following the procedures described by Niroomand‐Rad et al.^19^ and the three‐channel dosimetry approach for GRID therapy described by Gholami et al.^20^ Film measurements were cross‐checked against ion chamber and TPS doses over a range of dose levels, showing agreement within 2%. To better evaluate 3D lattice SFRT dose distributions, Gafchromic films were irradiated while sandwiched between solid water phantoms in both horizontal and vertical orientations. For all dosimetry methods, a 3%/1 mm gamma criterion with a 10% dose threshold and global normalization was used for gamma index analysis. For film dosimetry, in‐plane and cross‐plane dose profiles along the isocenter were compared with those calculated in the TPS. The lowest gamma passing rates across both in‐plane and cross‐plane profiles are reported in Table 1

**TABLE 1:** Dosimetric pass rates for EPID, film, and Delta4 using 3%/1 mm criteria for preliminary SFRT plans with varying vertex diameters and spacings, delivered on Edge (E) and Halcyon (H) linear accelerators.

Plan type	Vertex diameter	Vertex separation (mm)	Dose to vertices	SFRT GTV volume (cc)	MF (E/H)	Delta4 (E/H) (%)	Film (E/H) (%)	EPID (E/H) (%)
Single vertex	5 mm, 10 mm	30,40 (E)/ 35, 45 (H)	18 Gy × 1	64	2.5, 2.2 / 3.1, 3.0	100, 100 / 100, 100	96, 96 / 95, 95	100, 100 / 100, 100
Two vertices	5 mm, 10 mm	30, 40 (E)/ 35, 45 (H)	18 Gy × 1	177	2.6, 2.7 / 3.2, 2.9	100, 99 / 99, 100	97, 97/ 96, 94	100,100 / 100, 99
Three vertices	5 mm, 10 mm	30, 40 (E)/ 35, 45 (H)	18 Gy × 1	518	3.2, 2.8 / 4.1, 3.3	96, 99 / 99, 99	89, 90 / 91, 90	89, 93 / 96, 99
Seven vertices (1 plane)	5 mm, 10 mm	30, 40 (E)/ 35, 45 (H)	18 Gy × 1	518	5.7, 4.8 / 5.9, 4.9	96, 92 / 100, 96	86, 88 / 91, 90	90, 91 / 98, 95
15 vertices (3 planes)	5 mm, 10 mm	35, 45 (E)/ 40, 50 (H)	18 Gy × 1	700	4.6, 4.7 / 5.1, 4.9	95, 99 / 97, 99	89, 88 / 90, 90	90, 91 / 98, 99
Multiple targets (3 planes)	5 mm, 10 mm	40, 50 (E)/ 45, 55 (H)	18 Gy × 1	1153	4.3, 3.7 / 4.6, 3.8	96, 95 / 94, 94	89, 88 / 88, 88	90, 90 / 93, 93
Multiple targets (3 planes)	5 mm, 10 mm	40, 50 (E)/ 45, 55 (H)	66.7 Gy / 5 fx	1153	4.2, 3.6 / 4.4, 3.7	97, 99 / 100, 96	86, 88 / 86, 87	90, 93/ 93, 93

This combined approach, employing Delta4, EPID, and film dosimetry, enabled a more comprehensive assessment of the spatial dose characteristics of lattice SFRT.

## 3 RESULTS

**FIGURE 3:**
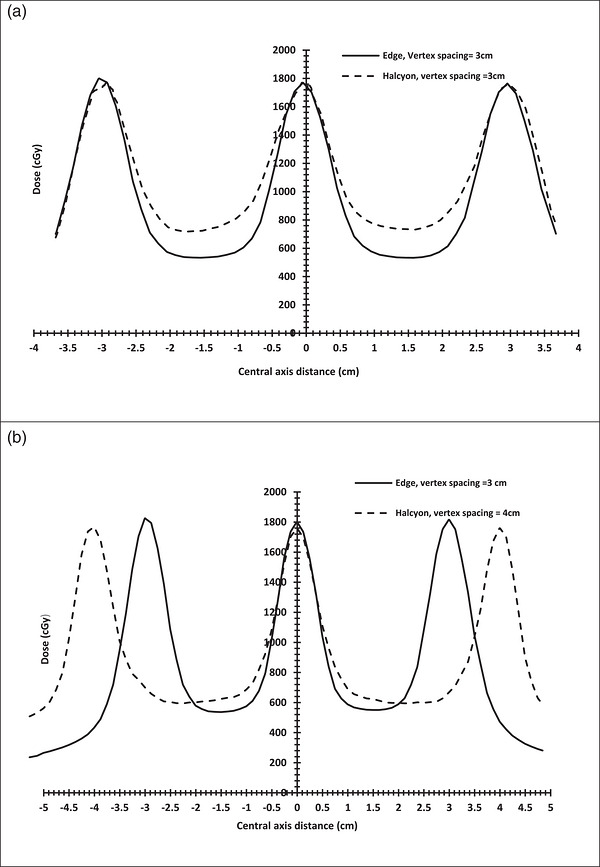
The TPS‐calculated dose profile comparison between Edge and Halcyon. (a) Identical vertex spacing (3 cm) used for both machines. (b) Increasing vertex spacing to 4 cm for Halcyon results in comparable PVDR.

Figure 3 (a) shows TPS‐calculated dose profiles for both Edge and Halcyon using identical vertex spacing (e.g., 3 cm), where the valley dose is higher for Halcyon. However, increasing the vertex spacing to 4 cm for Halcyon (Figure 3b) results in comparable point‐based PVDR (within ± 10%).

Table 1 summarizes the preliminary test geometries, including vertex diameter and separation, prescribed vertex dose, and the plan modulation factor (MF), defined as the ratio of monitor units (MU) to prescribed dose, along with corresponding dosimetry results from Delta4, film, and EPID measurements. In this table, the lowest pass rates were reported for film in both horizontal and vertical orientations across different fields, primarily due to uncertainties inherent to film dosimetry.^21^

**FIGURE 4:**
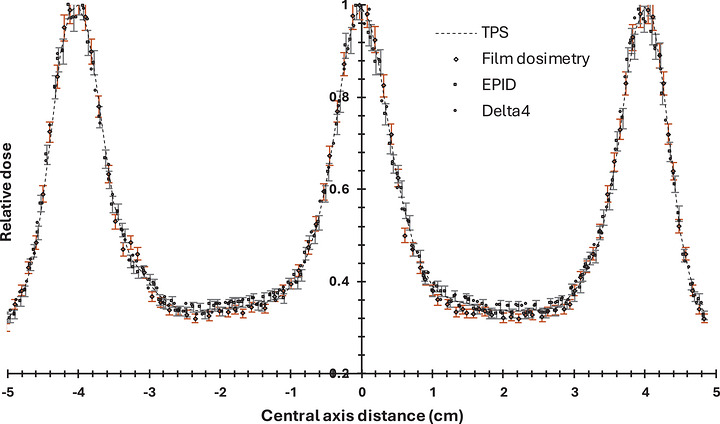
Representative comparison of TPS‐calculated and measured (film, EPID, Delta4) dose profiles for a vertex size of 0.5 cm and a spacing of 4 cm. Error bars represent ± 3%.

Figure 4 presents a representative comparison of TPS‐calculated cross‐plane dose profiles and corresponding measurements (film, EPID, and Delta4) from a “three‐vertex” test case with a vertex size of 0.5 and 4 cm spacing.

Table 2 summarizes the MF and the PVDR values across the six representative SFRT clinical test plans generated on Edge and Halcyon platforms. For the single‐fraction prescription (18 Gy in 1 fraction), MF values ranged from 3.6 to 4.4 for Edge and from 3.9 to 5.1 for Halcyon, with higher MF generally observed for larger SFRT‐GTV volumes, like pelvis–abdomen cases. For the SIB prescription (66.7 Gy & 20 Gy in 5 fractions), MF increased across all anatomical sites and for both platforms, reflecting the increased modulation required to achieve comparable lattice dose distributions at higher total dose. Despite these differences in MF, the point‐based PVDR values remained relatively consistent across treatment sites and platforms, ranging from approximately 2.8 to 3.4, indicating preservation of the intended spatial dose heterogeneity characteristic of SFRT. These results demonstrate that comparable lattice dose contrast can be achieved on both Edge and Halcyon systems, with platform‐ and prescription‐dependent differences in plan modulation complexity.

**TABLE 2:** Average modulation factor (MF) for six clinical SFRT test plans generated on Edge (E) and Halcyon (H) platforms for two prescription schemes (18 Gy in 1 fraction and 66.7 Gy in 5 fractions). The point‐based PVDR ranges (minimum–Maximum) for each prescription, derived from dose distributions in the axial, sagittal, and coronal planes across both platforms, are presented. DVH‐based PVDR values are reported separately for each platform (E/H) and prescription, along with the corresponding percentage differences (Δ) between platforms.

Plan	SFRT GTV volume	MF (18 Gy, E/H)	MF (66.7 Gy, E/H)	Point‐based PVDR (18 Gy)	Point‐based PVDR (66.7 Gy)	DVH‐based PVDR (18 Gy, E/H)	DVH‐based PVDR (66.7 Gy, E/H)
Head & neck	789.5 cc	3.6(E),3.9(H)	3.8(E),4.1 (H)	3.00‐3.21	3.10‐3.34	3.1(E), 3.04(H) (Δ∼1.9%)	3.24(E), 3.18(H) (Δ∼1.8%)
Thigh	810.2 cc	3.6(E),3.9(H)	5.5(E),5.6(H)	3.20‐3.30	3.11‐3.4	3.25(E), 3.20(H) (Δ∼1.5%)	3.31(E), 3.25(H) (Δ∼1.8%)
Pelvis‐abdomen	1632.8 cc	4.4(E),5.1(H)	5.7(E),6.3(H)	2.80‐3.10	2.90‐3.23	2.92(E), 2.88(H) (Δ∼1.4%)	3.02(E), 3.08(H) (Δ∼1.9%)

IMRT QA for all clinical test plans passed, with more than 90% of points meeting the 3%/1 mm gamma passing criterion using both EPID and Delta4. In cases with higher MF, the passing rate was higher for Delta4 than for EPID.

Table 3 summarizes the maximum values of the dose metrics (D0.03 cc, D1000 cc, or mean dose) to OARs for the evaluated clinical lattice plans.

**TABLE 3:** Maximum OAR dose metrics across all plans, including D0.03 cc, D1000 cc and mean dose, for clinical lattice plans delivered in single‐fraction (1 Fx) and SIB (5 Fx) regimens.

Structure	Lattice (1 Fx)	Lattice (5 Fx)
Optic nerves	D0.03 cc ≤ 400 cGy	D0.03 cc ≤ 1500 cGy
Brainstem	D0.03 cc ≤ 500 cGy	D0.03 cc ≤ 1500 cGy
Spinal cord	D0.03 cc ≤ 450 cGy	D0.03 cc ≤ 1300 cGy
Esophagus	D0.03 cc ≤ 500 cGy	D0.03 cc ≤ 1800 cGy
Brachial plexus	D0.03 cc ≤ 550 cGy	D0.03 cc ≤ 2000 cGy
Heart	D0.03 cc ≤ 500 cGy	D0.03 cc ≤ 1500 cGy
Great vessels	D0.03 cc ≤ 500 cGy	D0.03 cc ≤ 1500 cGy
Trachea	D0.03 cc ≤ 550 cGy	D0.03 cc ≤ 1500 cGy
Lung ‐GTV	D1000 cc≤ 500 cGy	D1000 cc≤ 1000 cGy
Stomach	D0.03 cc ≤ 600 cGy	D0.03 cc ≤ 1700 cGy
Duodenum	D0.03 cc ≤ 600 cGy	D0.03 cc ≤ 2000 cGy
Kidney_total	Mean ≤ 600 cGy	Mean ≤ 1000 cGy
Liver‐GTV	D700 cc≤ 300 cGy	D700 cc≤ 1250 cGy
Small bowel	D0.03 cc ≤ 600 cGy	D0.03 cc ≤ 1800 cGy
Large bowel	D0.03 cc ≤ 600 cGy	D0.03 cc ≤ 1700 cGy
Rectum	D0.03 cc ≤ 500 cGy	D0.03 cc ≤ 1800 cGy
Bladder	D0.03 cc ≤ 500 cGy	D0.03 cc ≤ 1900 cGy
Skin	D0.03 cc ≤ 500 cGy	D0.03 cc ≤ 2000 cGy

**FIGURE 5:**
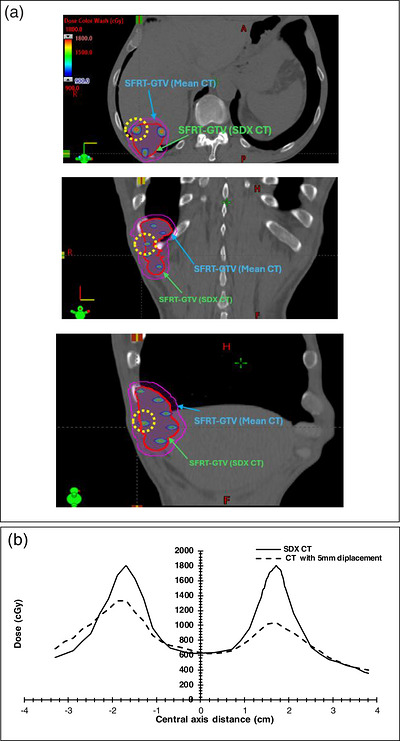
(a) Axial (top), coronal (middle), and sagittal (bottom) views of the SDX breath‐hold CT showing SFRT target structures. SFRT‐GTV and vertex structures contoured on the mean CT were transferred to the SDX CT. Yellow dashed regions indicate high‐dose vertices near or partially outside the SDX‐based SFRT‐GTV, highlighting potential increased dose to adjacent OARs due to respiratory‐induced anatomical differences. (b) Dose profile comparison along a line within the SFRT GTV on the SDX CT versus the dose profile along the same line with a 5 mm displacement in the superior–inferior direction.

Figure 5 (a) illustrates the spatial relationship of SFRT target structures on SDX breath‐hold CT images in the axial, coronal, and sagittal planes. The SFRT‐GTV and vertex structures, initially contoured on the mean CT, were rigidly transferred to the SDX CT to evaluate geometric fidelity. As shown, displacement of the anatomy relative to the planned lattice geometry can result in misalignment between the predefined vertices and the actual GTV position, with some high‐dose vertices shifting toward the target edge or partially outside the GTV (see yellow dashed line).

In the lung cases evaluated in this study, geometric mismatches directly impact the dose distribution within the target. Figure 5B) shows dose profile changes for a 5 mm displacement in the superior–inferior direction, resulting in a point‐based PVDR decrease of about 25%. Similar trends are observed for displacements in other directions (lateral and AP/PA). These effects indicate that motion can alter the vertex–valley structure, effectively smoothing the intended lattice pattern and potentially increasing the dose to adjacent OARs.

Table 4 summarizes the average OAR dosimetric metrics from respiratory motion management test plans using SDX across different scenarios, including mean CT with mean contours (without SDX), SDX CT with vertices contoured on the mean CT, and SDX CT with vertices contours derived from the corresponding SDX CT.

**TABLE 4:** Average values for OAR dosimetric metrics for SFRT plans across different scenarios with/without SDX motion management. Values are reported for Edge / Halcyon (E/H).

OARs	Mean CT with mean contours (E/H)	SDX CT with mean contours (E/H)	SDX CT with SDX contours (E/H)
Rib, D1cc	10.1 Gy/ 12.4 Gy	10.2 Gy/12.2 Gy	7.3 Gy/8.5 Gy
Lung, V 7 Gy	28.6 cc/ 35 cc	21.5 cc/ 32.7 cc	7.4 cc/8.1 cc
Liver, V 7 Gy	8.4 cc/ 9.9 cc	7.8 cc/ 9.1 cc	6.8 cc/8.0 cc
Skin, D 10cc	2.3 Gy/ 3.0 Gy	2.7 Gy /3.4 Gy	2.2 Gy/2.0 Gy

### 3.1 Dosimetric uncertainty

An estimate of the overall dosimetric uncertainty was considered for the key reported metrics. The main contributors include measurement uncertainties (film, Delta4, and EPID), dose calculation accuracy, and setup variability. The total uncertainty for film measurements was estimated at approximately 5%, considering both Type A and Type B components, including film calibration, scanning, and handling, in accordance with AAPM Task Group 138 and GEC‐ESTRO recommendations (DeWerd et al.).^22^

Detector‐based measurements (e.g., Delta4 and EPID) typically exhibit uncertainties on the order of 2%–3%, based on prior QA studies.^23^, ^24^ Additional uncertainties arise from spatial resolution limitations and positioning variability, particularly in high‐gradient regions.

Considering these combined effects, the overall uncertainty in vertex dose is estimated to be approximately 5%–6%, with slightly higher uncertainty in valley regions due to lower dose levels and increased sensitivity to spatial averaging. Consequently, the point‐based PVDR uncertainty across all three planes (axial, sagittal, and coronal) is estimated to be within ∼6%–10%.

## 4 DISCUSSION

This study demonstrates that a structured pathway supports the reproducible clinical implementation of VMAT‐based lattice SFRT across two distinct linac platforms (Edge TrueBeam and Halcyon). By progressing from phantom‐based studies—ranging from single‐vertex to multi‐plane, high‐vertex‐count configurations—to site‐specific clinical test plans, we established practical planning and verification procedures that maintained the intended spatial dose heterogeneity while meeting measurement‐based QA tolerances. Across all clinical tests, the point‐based PVDR ranged from 2.8–3.3 for a prescription of 18 Gy at the peak and 6 Gy at the valley, and from 2.9–3.4 for a prescription of 66.7 Gy at the peak and 20 Gy at the valley (in 5 fractions). The corresponding variations in point‐based peak and valley doses relative to planned values were within approximately ± 10%, which is consistent with measurement and sampling uncertainties in high‐gradient regions. These results indicate that the lattice dose structure was preserved despite differences in MLC design between the two machines.

Our findings confirm that both platforms can generate clinically feasible lattice plans with comparable PVDR performance. However, due to the larger leaf width of the Halcyon MLC (5 mm vs. 2.5 mm at isocenter), achieving similar dose fall‐off and spatial distribution may require increased inter‐vertex spacing and higher plan modulation. This is consistent with our observation that the MF increases with larger SFRT‐GTV volumes.

Preliminary phantom tests revealed a key trade‐off in lattice planning: smaller vertices result in sharper dose gradients (and potentially higher PVDR) but also increase sensitivity to small‐field modeling, MLC positioning limitations, and measurement uncertainty. In our workflow, vertex diameters of 0.5–1.0 cm with center‐to‐center spacing of 3–5.5 cm and inter‐plane spacing of approximately 1.5–2 cm provided a practical balance between peak localization and plan robustness, while maintaining a low vertex‐to‐target volume ratio and reducing the risk of placing high‐dose vertices near the SFRT‐GTV boundary. These findings align with previously published work demonstrating that vertex sizes in the sub‐ to low‐centimeter range and spacings of several centimeters yield favorable peak‐to‐valley dose characteristics in lattice radiotherapy planning.^25^, ^26^ However, our suggested systematic workflow further enhanced understanding of contouring requirements specific to machine MLC type and GTV volume across treatment sites before clinical SFRT implementation.

Our observation indicates that for larger targets (e.g., pelvis–abdomen), increasing vertex diameter and spacing improved plan stability and reduced unnecessary modulation without compromising PVDR. This is consistent with our finding that PVDR remained relatively stable across large‐volume clinical tests. Larger vertices (1–1.5 cm) produced more stable QA results and were easier to optimize, particularly for large targets, but at the cost of reduced PVDR. Similar results were reported by Zhang et al.,^27^ reinforcing the importance of tailoring vertex size to tumor geometry, location, and treatment intent. Additionally, planning for large pelvic or abdominal volumes may require two isocentric plans due to machine collimator size limitations. On the other hand, for head‐and‐neck treatments, using smaller vertex diameters (e.g., 0.5 cm) resulted in higher modulation, increasing sensitivity to calculation resolution and delivery uncertainties. However, since these lesions are typically smaller than those in pelvic or abdominal sites, the smaller vertex size allowed for more vertices within the target volume, increasing the number of peak‐to‐valley dose regions within the SFRT‐GTV. Our findings are consistent with those of Wu et al.,^8^ who observed that smaller vertices achieved sharper dose gradients and higher PVDR values, which is advantageous for smaller targets such as head and neck tumors where OAR sparing is critical.

These findings suggest that a site‐ and volume‐adaptive vertex design is preferable to a single fixed lattice template.

For moving targets such as lung and liver tumors, treatment planning based on the mean CT from 4D CT may introduce interplay effects due to respiratory motion. Since the ITV is larger than the instantaneous GTV, a greater number of lattice vertices may be placed within the ITV. As a result, respiratory motion can lead to displacement of lattice vertices and valley regions, potentially causing high‐dose vertices to overlap with adjacent OARs (e.g., skin or ribs) or extend outside the intended SFRT‐GTV, as illustrated in Figure 5.

In the evaluated lung scenario, displacements on the order of 5 mm can result in misalignment of vertex positions, degradation of point‐based PVDR (approximately 25%), and alteration of the dose distribution due to interplay effects and dose blurring. Therefore, the impact of motion depends on the magnitude of displacement relative to vertex size and spacing and the distance of vertices from OARs, rather than a single fixed threshold.

A more detailed and quantitative evaluation of motion effects is recommended for each institution to optimize SFRT planning and implementation for moving targets. Motion management strategies are therefore important to maintain vertex positioning and preserve the intended lattice dose distribution.

Table 4 demonstrates clinically relevant changes in OAR metrics when the plan/vertices defined on mean CT were propagated to the SDX dataset. The direction and magnitude of these differences (e.g., rib D1cc, lung V7 Gy, and skin D10cc reductions when using SDX‐derived contours) underscore that motion‐managed imaging and target definition can significantly improve OAR sparing and reduce unintended peripheral high‐dose placement. Our findings are consistent with those of Martin‐Paulpeter et al.,^28^ who found that motion management strategies such as gating or breath‐hold are necessary to preserve PVDR, though these may increase workflow complexity and treatment time.

Because lattice SFRT intentionally creates steep internal dose gradients, verification should prioritize: (a) the geometric positioning of peak and valley regions and (b) overall agreement using criteria appropriate for highly modulated fields. Film dosimetry is the preferred method for dose verification due to its superior spatial resolution for evaluating peak and valley structures. However, consistent with the uncertainty estimates used in this study, it exhibited lower gamma pass rates for 3%/1 mm criteria with a 10% dose threshold, owing to the combined uncertainties of film calibration, scanning, and analysis (approximately 5%).^29^ Delta4 met acceptance for both preliminary and clinical plans on both machines, frequently achieving high pass rates even when EPID pass rates approached the lower acceptance boundary. This aligns with previous reports indicating that diode arrays are better suited for complex dose distributions, whereas EPID‐based QA may fail due to limitations of MV imaging panels, including dose‐rate saturation and energy dependence in FFF beams.^24^, ^29^

Practically, these findings support a tiered QA strategy: (a) use a high‐density detector array (e.g., diode array) as the primary quantitative QA tool for routine clinical implementation, and (b) reserve film dosimetry for commissioning, troubleshooting, and periodic verification of peak–valley integrity, rather than for routine accept/reject decisions.

In addition, it is important to note that dose calculation accuracy in lattice SFRT is challenged by small field sizes and steep dose gradients.^30^ Although several studies have evaluated the accuracy of Acuros XB for small‐field dosimetry,^31^, ^32^ Monte Carlo (MC) methods remain the gold standard for SFRT dose calculations.^33^ Therefore, small discrepancies between calculated and measured doses are expected, particularly in sub‐centimeter fields and high‐gradient regions. Dose grid resolution is also critical, as SFRT dose distributions are highly sensitive to voxel size (1–1.25 mm grid size was used in this study). Coarser grids can introduce volume‐averaging effects and lead to underestimation of PVDR.

Although the proposed framework is described as platform‐agnostic, both implementations in this study were performed within a single treatment planning system (Eclipse). Therefore, the generalizability of the results to other planning systems may be limited. Differences in dose calculation algorithms, optimization engines, beam energies (e.g., 6 MV FFF vs. 10 MV FFF), MLC characteristics, and grid resolution across TPS platforms could influence lattice dose distributions, vertex definition, and PVDR metrics.

Nevertheless, the underlying principles of the proposed workflow, including vertex placement strategy, dose prescription, and evaluation metrics (e.g., PVDR and dose profiles), are not inherently dependent on a specific TPS and can be adapted to other systems. However, quantitative differences in dosimetric outcomes may arise when implemented in different TPS environments.

In summary, the findings of this study should be interpreted as validation of an implementation methodology, rather than a prescription of fixed planning parameters or expected dosimetric outcomes. Variations in lattice geometry, PVDR, and QA metrics are expected due to user‐ and system‐dependent factors, including MLC resolution, optimization choices, and prescription intent. The structured progression of tests presented here is intended to support institutional learning and commissioning, enabling centers to characterize their own planning and delivery limits prior to clinical use. In this context, the strength of this work lies in the reproducibility of the process, not the exact numerical results.

## 5 CONCLUSION

This study demonstrates a practical, method‐based framework for implementing lattice SFRT across the evaluated linac platforms (Edge/TrueBeam and Halcyon) through a structured progression from phantom testing to representative clinical scenarios. By emphasizing planning parameter selection, measurement‐based QA, and site‐specific validation, the framework enables institutions to develop lattice SFRT plans that depend on the treatment site and the available machine type.

Our future work will focus on further investigating respiratory motion control strategies for lattice SFRT in a larger number of moving targets to better define best practices for thoracic and abdominal applications. In addition, broader validation through multi‐institutional studies using a similar implementation method will support improved reproducibility and facilitate the development of consensus guidance for clinical lattice SFRT adoption.

## AUTHOR CONTRIBUTIONS

**Somayeh Gholami**: Conceptualization; methodology; software; analysis; investigation; writing—original draft; supervision. **Prema Rassiah**: Supervision; analysis; writing—review and editing.

## CONFLICT OF INTEREST STATEMENT

The authors declare no conflicts of interest.

## ETHICAL APPROVAL

Ethical review and approval were waived for this study because it involved only phantom data and fully de‐identified retrospective clinical plans, with no human subjects or identifiable patient information.

## Data Availability

The data supporting this study are available from the corresponding author upon reasonable request.

## References

[1] Fernandez‐Palomo C , Chang S , Prezado Y . Should peak dose be used to prescribe spatially fractionated radiation therapy?–a review of preclinical studies. Cancers. 2022;14:3625. doi:10.3390/cancers14153625 35892895 PMC9330631

[2] Mohiuddin M , Fujita M , Regine WF , Megooni AS , Ibbott GS , Ahmed MM , High‐dose spatially‐fractionated radiation (GRID): a new paradigm in the management of advanced cancers. Int J Radiat Oncol Biol Phys. 1999;45(3):721‐727. doi:10.1016/S0360-3016(99)00170-4 10524428

[3] Gholami S , Nedaie HA , Longo F , Ay MR , Wright S , Meigooni AS . Is grid therapy useful for all tumors and every grid block design?. J Appl Clin Med Phys. 2016;17(2):206‐219. doi:10.1120/jacmp.v17i2.6015 27074484 PMC5874944

[4] Gholami S , Nedaie HA , Longo F , Ay MR , Dini SA , Meigooni AS . Grid block design based on monte carlo simulated dosimetry, the linear quadratic and Hug–Kellerer radiobiological models. J med phys. 2017;42(4):213‐221. 29296035 10.4103/jmp.JMP_38_17 PMC5744449

[5] Pokhrel D , Halfman M , Sanford L , Chen Q , Kudrimoti M . A novel, yet simple MLC‐based 3D‐crossfire technique for spatially fractionated GRID therapy treatment of deep‐seated bulky tumors. J Appl Clin Med Phys. 2020;21(3):68‐74. doi:10.1002/acm2.12826 PMC7075376 32034989

[6] Jin J‐Y , Zhao B , Kaminski JM , et al. A MLC‐based inversely optimized 3D spatially fractionated grid radiotherapy technique. Radiother Oncol. 2015;117(3):483‐486. doi:10.1016/j.radonc.2015.07.047 26277434

[7] Owen D , Harmsen WS , Ahmed SK , et al. Highs and lows of spatially fractionated radiation therapy: dosimetry and clinical outcomes. Pract Radiat Oncol. 2025;15(4):e388‐e395. doi:10.1016/j.prro.2024.12.002 39725127

[8] Wu X , Perez NC , Zheng Y , et al. The technical and clinical implementation of LATTICE radiation therapy (LRT). Radiat Res. 2020;194(6):737‐746. doi:10.1667/RADE-20-00066.1 33064814

[9] Li H , Mayr NA , Griffin RJ , et al. Overview and recommendations for prospective multi‐institutional spatially fractionated radiation therapy clinical trials. Int J Radiat Oncol Biol Phys. 2024;119(3):737‐749. doi:10.1016/j.ijrobp.2023.12.013 38110104 PMC11162930

[10] Grams MP , Deufel CL , Kavanaugh JA , et al. Clinical aspects of spatially fractionated radiation therapy treatments. Physica Med. 2023;111:102616. doi:10.1016/j.ejmp.2023.102616 37311338

[11] Kim MM , Bollinger D , Kennedy C , et al. Dosimetric characterization of the dual layer MLC system for an O‐ring linear accelerator. Technol Cancer Res Treat. 2019;18:1533033819883641. doi:10.1177/1533033819883641 31707918 PMC6843729

[12] Ojala J . The accuracy of the Acuros XB algorithm in external beam radiotherapy–a comprehensive review. Int J Cancer Ther Oncol. 2014;2(4):020417. doi:10.14319/ijcto.0204.17

[13] Timmerman RD . An overview of hypofractionation and introduction to this issue of seminars in radiation oncology. Semin Radiat Oncol; 2008;18(4):215‐222. 18725106 10.1016/j.semradonc.2008.04.001

[14] Burns L , Tsai J , Wong P , et al. Spatially fractionated radiotherapy for re‐irradiation: feasibility, safety, treatment planning, and outcomes. Clin Transl Radiat Oncol. 2025;56:101049. 41080985 10.1016/j.ctro.2025.101049 PMC12512144

[15] Amendola BE , Perez NC , Mayr NA , Wu X , Amendola M . Spatially fractionated radiation therapy using lattice radiation in far‐advanced bulky cervical cancer: a clinical and molecular imaging and outcome study. Radiat Res. 2020;194(6):724‐736. doi:10.1667/RADE-20-00038.1 32853384

[16] Misa J , Pokhrel D . Demonstration of an enhanced dosing pattern for debulking large and bulky unresectable tumors via differential hole‐size spatially fractionated radiotherapy. J Appl Clin Med Phys. 2025;26(7):e70127. doi:10.1002/acm2.70127 40421508 PMC12256665

[17] Pakela JM , Knopf A , Dong L , Rucinski A , Zou W . Management of motion and anatomical variations in charged particle therapy: past, present, and into the future. Front Oncol. 2022;12:806153. doi:10.3389/fonc.2022.806153 35356213 PMC8959592

[18] Darafsheh A , Ghaznavi H . A review on radiochromic film dosimetry in radiation therapy. J Appl Clin Med Phys. 2025;26(12):e70365. doi:10.1002/acm2.70365 41330745 PMC12672139

[19] Niroomand‐Rad A , Chiu‐Tsao ST , Grams MP , et al. Report of AAPM task group 235 radiochromic film dosimetry: an update to TG‐55. Med Phys. 2020;47(12):5986‐6025. doi:10.1002/mp.14497 32990328

[20] Gholami S , Nedaie HA , Meigooni AS . Precise EBT3 Gafchromic film dosimetry for Grid therapy. Radiat Meas. 2019;121:69‐76. doi:10.1016/j.radmeas.2018.11.008

[21] Bouchard H , Lacroix F , Beaudoin G , Carrier JF , Kawrakow I . On the characterization and uncertainty analysis of radiochromic film dosimetry. Med Phys. 2009;36(6Part1):1931‐1946. doi:10.1118/1.3121488 19610282

[22] DeWerd LA , Ibbott GS , Meigooni AS , et al. A dosimetric uncertainty analysis for photon‐emitting brachytherapy sources: report of AAPM Task Group No. 138 and GEC‐ESTRO. Med Phys. 2011;38(2):782‐801. doi:10.1118/1.3533720 21452716 PMC3033879

[23] Bedford JL , Lee YK , Wai P , South CP , Warrington AP . Evaluation of the Delta4 phantom for *IMRT and VMAT verification* . Phys Med Biol. 2009;54(9):N167‐N176. doi:10.1088/0031-9155/54/9/N04 19384007

[24] Dogan N , Mijnheer BJ , Padgett K , et al. AAPM task group report 307: use of EPIDs for patient‐specific IMRT and VMAT QA. Med Phys. 2023;50(8):e865‐e903. doi:10.1002/mp.16536 37384416 PMC11230298

[25] Seol Y , Lee YK , Kim BJ , et al. Feasibility of optimal vertex size and spacing for lattice radiotherapy implementation using helical tomotherapy. Front Oncol. 2025;15:1512064. doi:10.3389/fonc.2025.1512064 40171269 PMC11959701

[26] Lee YK , Seol Y , Kim BJ , et al. A preliminary study of linear accelerator‐based spatially fractionated radiotherapy. Front Oncol. 2025;14:1495216. doi:10.3389/fonc.2024.1495216 39876900 PMC11772434

[27] Zhang X , Griffin RJ , Galhardo EP , Penagaricano J . Feasibility study of 3D‐VMAT‐based GRID therapy. Technol Cancer Res Treat. 2022;21:15330338221086420. doi:10.1177/15330338221086420 35289202 PMC8928354

[28] Martin‐Paulpeter RM , Balter PA , Perles LA , Ludmir EB , Niedzielski JS . Respiratory motion effects and plan robustness for lattice radiation therapy. Front Oncol. 2026;16:1731981. doi:10.3389/fonc.2026.1731981 41821887 PMC12975452

[29] Xu Q , Huynh K , Nie W , et al. Implementing and evaluating a high‐resolution diode array for patient‐specific quality assurance of robotic brain stereotactic radiosurgery/radiotherapy. J Appl Clin Med Phys. 2022;23(5):e13569. doi:10.1002/acm2.13569 35278033 PMC9121027

[30] Das IJ , Khan AU , Dogan SK , Longo M . Grid/lattice therapy: consideration of small field dosimetry. Br J Radiol. 2024;97:1088‐1098. doi:10.1093/bjr/tqae060 38552328 PMC11135801

[31] Sarin B , Bindhu B , Kumar PR , Sumeesh S , Saju B . Dosimetric accuracy of Acuros® XB and AAA algorithms for stereotactic body radiotherapy (SBRT) lung treatments: evaluation with PRIMO Monte Carlo code. J Radiother Pract. 2023;22:e65. doi:10.1017/S1460396922000346

[32] Lárraga‐Gutiérrez JM , García‐Garduño OA , Herrera‐González JA , de la , Cruz OOG . Evaluation of Acuros® XB accuracy for static small fields dose calculations based on the IAEA/AAPM TRS‐483 recommendation. Physica Med. 2021;89:140‐146. doi:10.1016/j.ejmp.2021.06.021 34365118

[33] Zhang H , Mayr NA . Spatially Fractionated, Microbeam and FLASH Radiation Therapy: A Physics And Multi‐Disciplinary Approach. IOP Publishing; 2023.

